# Self-managed physical activity in cancer survivors for the management of cancer-related fatigue: A scoping review

**DOI:** 10.1371/journal.pone.0279375

**Published:** 2022-12-21

**Authors:** Isabella Campanini, Maria Bernadette Ligabue, Maria Chiara Bò, Maria Chiara Bassi, Mirco Lusuardi, Andrea Merlo

**Affiliations:** 1 LAM - Motion Analysis Laboratory, Neuromotor and Rehabilitation Department, San Sebastiano Hospital, Azienda USL-IRCCS di Reggio Emilia, Correggio, Reggio Emilia, Italy; 2 Motor Rehabilitation Unit, Neuromotor and Rehabilitation Department, San Sebastiano Hospital, Azienda USL-IRCCS di Reggio Emilia, Correggio, Reggio Emilia, Italy; 3 Merlo Bioengineering, Parma, Italy; 4 Medical Library, Azienda USL-IRCCS di Reggio Emilia, Reggio Emilia, Italy; 5 Neuromotor and Rehabilitation Department, Azienda USL-IRCCS Reggio Emilia, Reggio Emilia, Italy; Universiteit Antwerpen, BELGIUM

## Abstract

**Objective:**

Cancer-related fatigue (CRF) is a disabling chronic condition that cancer survivors could experience during and after recovery and that might benefit from self-managed physical activity (PA) programs. This scoping review aimed to map self-managed PA interventions found in literature for the management of CRF.

**Methods:**

Given the heterogeneity of the topic, scoping review methodological frameworks were used. Pubmed, Cinahl and Cochrane databases were searched for primary literature. Inclusion criteria: self-managed PA meant as any exercise program prescribed by a professional either with or without initial supervision and training which then continued independently for a given time frame; patient-reported fatigue assessment included in the outcome measures. Articles dealing with entirely supervised interventions, dietary or psychological-only therapies, and with palliative care were excluded.

**Results:**

Of the 543 experimental or observational studies screened, 63 were included. Of these forty-three studies were randomized controlled trials. Data were summarized in tables describing self-managed interventions according to: type of self-managed activity, frequency and duration, strategies to promote adherence, professionals supervising the treatment, outcome measures, and efficacy. A narrative synthesis was also added to further explain findings.

**Conclusions:**

We collected the available evidence on PA when this was self-managed by patients after prescription by a healthcare provider. Clinicians and researchers should consider incorporating self-care programs in CRF patients’ recovery journey gradually, identifying the best strategies to integrate them into daily life. Researchers should specify the characteristics of PA programs when designing new studies. This review highlighted the areas to be investigated for future studies pertaining to self-managed PA.

## Introduction

Cancer is the worldwide leading cause of death, with more than 18 million new cases diagnosed each year. The most common cancer sites are breast, colorectal, lung, prostate, skin, and bladder, with survival rates varying considerably [1]. The incidence of this disease is estimated to increase significantly in the coming decades, associated with an ever-increasing survival rate also thanks to progress in modern medicine and early screening programs [1, 2].

The cancer therapies that patients undergo and the aggressiveness of the disease itself can lead to the onset of side effects that affect the biopsychosocial functioning of cancer survivors (CS), limiting their return to social, working and leisure activities [3]. Cancer-related fatigue (CRF) is a frequent complex symptom in CS, which may persist even several years after the end of a treatment [4]. In patients it manifests as a persistent and subjective sense of exhaustion, affecting their physical, emotional, and mental spheres, and the fatigue perception is not compatible to the activity being performed and cannot be alleviated by rest or sleep [5]. This outcome affects the quality of life of both patients and their caregivers, and put in question whether it is worth continuing the oncological therapies due to the side effects, consequently hindering the healing process and their recovery chances [4, 6]. Since the etiology of CRF seems to be multifactorial, several approaches have attempted to control it, such as pharmacological therapies, different types of physical activity (PA), diet, sleep therapies and psychological support [4]. Among these, exercising led to promising results, along with psychological interventions, especially when delivered in-person, while pharmacological therapies did not [7].

Given the increase in survival rates, cancer is now increasingly considered a chronic condition [8, 9]. The same could be said specifically of CRF, as it can persist for many years after the completion of the therapies [4]. The Chronic Care Model, designed by Wagner in 1998, stated that healthcare systems must manage chronic diseases by teaching patients self-management techniques, building self-esteem and symptom-control skills. This way, patients refer to clinicians only at the planned follow-ups or, exceptionally, when their symptoms flare up and when the management treatment needs to be adapted or updated [10]. However, self-managed PA programs are still difficult to implement, due to multiple factors, including suitable patient training and self-awareness and the tendency of dropping out of said programs when patients are unsupervised. Most importantly this self-care must become an integral part of a patient’s daily routine [11].

Since the topic of self-managed rehabilitation for CRF is still heterogenous, a scoping review is a suitable tool in order to analyze it in its entirety. Scoping reviews map all available evidence on a broad-ranging topic, examining how research is conducted and highlighting the gaps in knowledge in a field that is constantly evolving [12, 13]. Unlike systematic reviews, scoping reviews do not intend to answer either specific or restrictive questions by appraising and synthesizing the results in literature [14].

Existing systematic reviews on the effects of PA on CRF [6, 15–17] include studies using both self-managed and fully supervised programs in their analyses. Consequently, the effect of self-managed PA remains unclear. Given the previously demonstrated effectiveness of supervised exercise in reducing and controlling CRF [6, 15], this scoping review aim is to investigate all available characteristics found in literature regarding self-managed rehabilitation pathways recommended for reducing or controlling CRF in CS. We were especially interested in programs involving PA as the primary intervention. Having a clearer understanding of existing literature on this new topic will allow for appropriate efficacy studies to be designed and conducted in the future.

## Methods

We followed the guidelines specific for scoping reviews [12], which are an extension of the PRISMA guidelines for systematic review [18]. The methodology consists of a six-stage process, described below.

### Stage 1: Identifying the research question

The leading questions of this investigation were: I) Which types of self-managed PA are used to treat CRF in CS? II) How is self-care delivered?—What is the duration and the frequency necessary to counteract the symptoms? III) What effects are obtained? IV) Which strategies have been employed to help patients embrace self-managed activities in their daily life?

### Stage 2: Identifying relevant studies

Comprehensive and systematic searches were devised and tuned also with the help of a specialist at our Institution. In February 2022 an investigation of the following databases was conducted: Medline, Cinahl, and Cochrane databases. No time limitations were imposed and only articles published in English or Italian were considered, depending on the language skills of the authors.

Keywords searched included:“cancer”,“tumor”,“cancer survivors”,“self-care”,“physical therapy modalities”,“rehabilitation”,“exercise”,“home-based care”, and“fatigue”. When available, medical Subjects Headings (MeSH) were included to ensure consistency of search terms. The complete search strategies can be consulted in Table 1. Papers not retrieved by the search string were added by manually searching the references of the included studies.

**Table 1:** Search strategies of the searched databases.

Database	Strategy
**Medline (PubMed)**	(“Cancer Survivors”[Mesh] OR cancer survivor*) AND (“Self Care”[Mesh] OR self cure OR self care OR self management OR home-based OR unsupervised) AND (“Physical Therapy Modalities”[Mesh] OR rehabilitation OR physical therap* OR physiotherapy OR exercise* OR physical activity) AND (“Fatigue”[Mesh] OR cancer-related fatigue OR fatigue OR crf)
**Cochrane Database**	(MeSH descriptor: [Cancer Survivors] explode all trees OR cancer survivor*) AND (MeSH descriptor: [Self Care] explode all trees OR self cure OR self care OR self management OR home-based OR unsupervised) AND (MeSH descriptor: [Physical Therapy Modalities] explode all trees OR rehabilitation OR physical therap* OR physiotherapy OR exercise* OR physical activity) AND (MeSH descriptor: [Fatigue] explode all trees OR cancer-related fatigue OR fatigue OR crf)
**Cinahl Database**	((MH "Cancer Survivors" OR survivor*)) AND (MH "Combined Modality Therapy" OR MH "Physical Therapy" OR rehabilitation OR physical therap* OR physiotherapy OR exercise* OR home based care)) AND (MH "Self Care" OR self cure OR self care OR self management OR OR home-based OR unsupervised) AND (MH "Combined Modality Therapy" OR MH "Physical Therapy" OR rehabilitation OR physical therap* OR physiotherapy OR exercise*) AND (MH "fatigue" OR cancer-related fatigue OR fatigue OR crf)

### Stage 3: Selecting studies

#### Types of studies

We included primary quantitative literature, such as experimental or observational studies that involved a clear self-managed activity prescribed by a professional. We excluded: 1) studies examining only the prevalence of CRF or self-initiated activities with cross-sectional study designs; 2) qualitative studies; 3) secondary literature, such as reviews and meta-analyses; 4) pilot studies for which a corresponding broader randomized trial was available, since they would have been duplicates, and 5) any literature where the full text was not available.

#### Population

CS adults were the population of interest. Since the term cancer survivor refers to anyone who has ever been diagnosed with cancer, regardless of the stage of their disease [19], we included studies with both CS who had completed their primary therapies and patients who were still undergoing treatment. Papers including patients at advanced stages of the disease only requiring palliative care and papers on children were excluded.

#### Intervention

In this study, the adjective “self-managed” refers to any exercise program that was prescribed by a professional either with or without initial supervision and training which then continued independently for a given time frame (at least one month), before the patient was evaluated again at follow-up appointments. According to the aim of this study, we focused on the results of the self-managed phase only. The duration of the initial supervised training, when delivered, was reported in the S1 Table and later discussed in this paper.

Studies that offered in-person support during the self-management phase were considered eligible only if meetings were scheduled no more than every 2 weeks. Strategies adopted during the self-managed period including remote support (e.g. phone calls or text messages involving behavioral changes techniques and periodic checks) were considered “mild” supervision as these excluded both in-person visits and comprehensive revision of patients’ behaviors. In this case, any PA performed without an in-person support by healthcare professionals, was therefore considered as a fully self-managed activity. For this reason, no frequency or time limits were set for remote support when considering whether to include a study. We excluded studies offering interventions such as dietary or psychological therapies without a PA component, and studies providing exclusively supervised sessions.

#### Comparison

The intervention could be compared with any type of control.

#### Outcome

The outcome measure had to be self-reported and related to CRF. Only measurements related to the self-managed phase were reported. Studies that only explored complementary aspects of CRF, such as functional capacity, without directly investigating fatigue with appropriate scales were excluded.

#### Context

No limitations were set regarding the context of the studies (e.g. gyms, other sports facilities, and home-based exercising).

### Stage 4: Charting the data

Two reviewers (AM and MCBO) screened all articles and assessed them for inclusion according to the criteria detailed in Stage 3. Any discrepancy was solved by involving a third reviewer (IC). Relevant data were extracted from each study and collected in tables. To maximize the accuracy of extractions, authors had ongoing discussions pertaining to any relevant changes being made to the tables.

### Stage 5: Summarizing and reporting the data

Information about study design, type of exercise, duration and frequency of an intervention, and the strategies used to help CS keep exercising in the long term, as well as outcome measures and reported effectiveness were extracted from each study and organized into tables. Additionally, we collected secondary outcomes related to exercise tolerance and physical ability when present, as indicators of exercise-related fatigue and thus equally relevant in the patients’ return to a disease-free life. Findings were also presented in a narrative synthesis.

### Stage 6: Consultation

A CRF oncological rehabilitation expert (MBL) was contacted and consulted throughout the planning and execution of the study to help establish the best inclusion criteria and in identifying all relevant studies that could have been missed. Final findings were also discussed.

## Results

**Fig 1:**
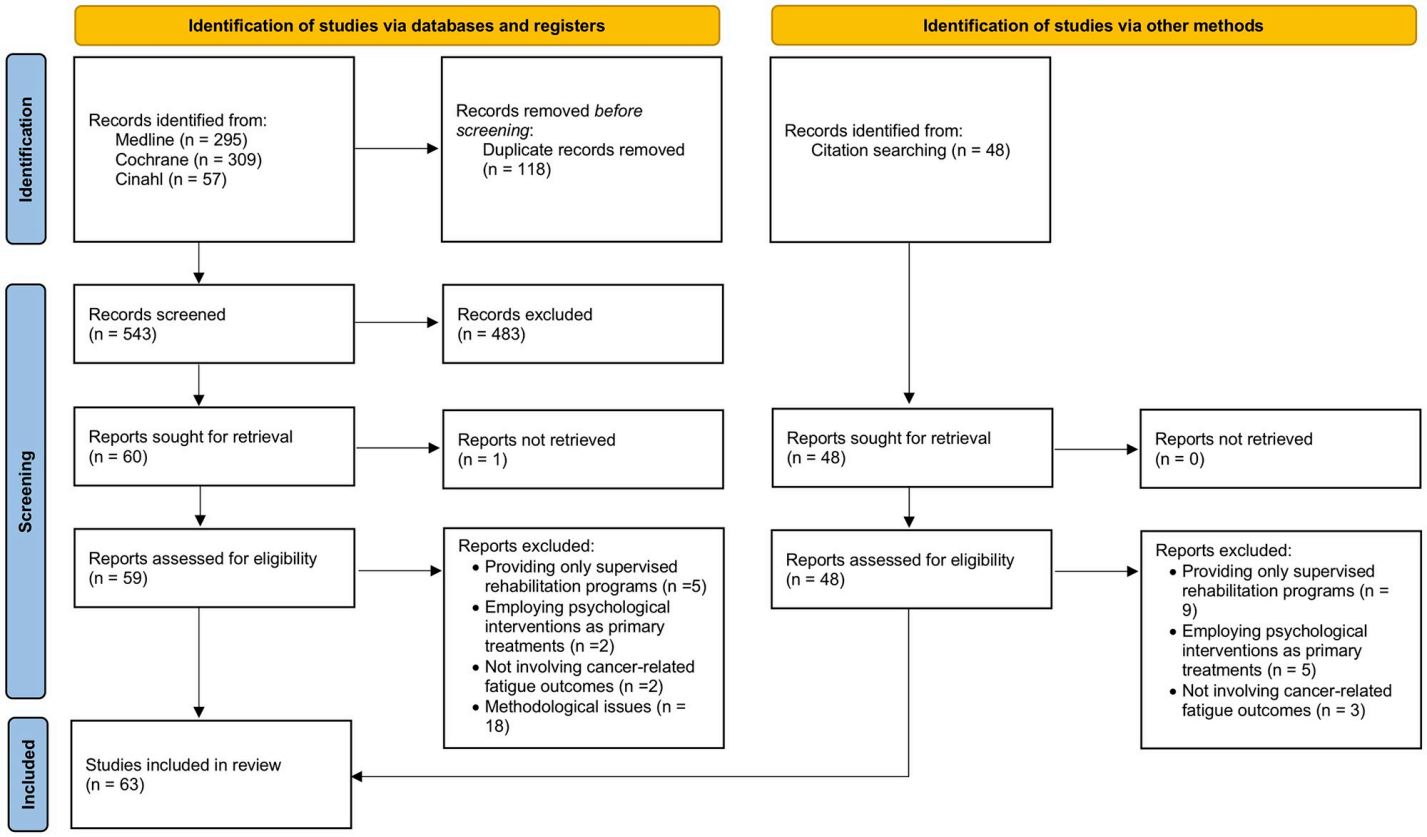
Flow chart of the literature search on self-managed physical activities for cancer-related fatigue management.

The literature search led to the identification of 661 studies. After the removal of duplicates, 543 studies were screened by title and abstract, and 59 were selected for full-text analysis. Of these, 32 were included in the final manuscript, to which we added 31 studies retrieved from hand searching relevant references of the included studies [20–82], leading to a total of 63 articles. The Flow Chart of the study selection is presented in Fig 1.

Complete results of the search are reported in the S1 Table. Out of the 63 included studies, 68% (43/63) were randomized controlled trials (RCT), 16% (10/63) had a single-arm design, 13% (8/63) were pilot studies, and 3% (2/63) were non-randomized clinical trials. Among these studies the sample size ranged from 9 to 360 participants. Ten studies had no control group. Fifty-three studies were compared to at least one control group, as shown in Table 2.

**Table 2:** Included control groups.

Type of control group	Number of studies
*Two-arm studies (n = 48)*	
Active control group	7
Educational control group	5
Similar activity with supervision	1
Usual care	35
*Three-arm studies (n = 5)*	
Active control group and usual care	3
Educational control group and usual care	1
Similar activity with supervision and usual care	1
*Without control group (n = 10)*	

Two studies compared two self-managed treatments to a common control group [49, 80]. Consequently, these were inserted twice in the following tables, leading to a total of 65 mapped interventions (as illustrated in Table 3). Forty-three out of sixty-five (66%) interventions addressed breast cancer survivors, three interventions (5%) addressed both survivors from gynecological cancers or lymphoma, two interventions (3%) addressed colorectal, lung cancer or myeloma survivors. Ten out of sixty-five (15%) treatments were provided to a mixed sample of cancer survivors.

**Table 3:** Summary of self-managed interventions: Type of activity, duration, recommended frequency, and professional in charge of managing cancer survivors.

Characteristics of the interventions	Number of interventions (n = 65)
*Type of activity*	
Aerobic exercise program	28
Resistance exercise program	1
Combined exercise program (aerobic, resistance, stretching, flexibility)	19
Mind-body practice (yoga, tai chi, qigong)	10
Multidimensional program (physical, nutritional, psychological)	7
*Duration*	
1–2 months	20
3–5 months	28
6–11 months	13
≥ 1 year	4
*Frequency*	
1–2 times / week	6
≥ 3 times / week	38
Daily	7
Not available	14
*Responsible professional*	
Exercise expert	13
Multidisciplinary team	11
Physiotherapist	10
Mind-body teacher	6
Nurse	5
Other	2
Not specified	18

Details of self-managed activities in terms of the type of exercise, dosage (duration and weekly frequency), and the professional in charge of the patient management are presented in Table 3. We also investigated whether researchers facilitated patients in the gradual acquisition of self-managed activities into a daily routine, mapping the conduct of any supervised sessions before the period of autonomy–most of them included gradually introducing “homework assignments” and independent exercising–and the strategies adopted to help maintain long-term treatment adherence, as illustrated in Table 4.

**Table 4:** Initial supervision to the interventions and strategies to increase cancer survivors’ assimilation of self-management in their routine.

Help to implement self-management into the daily routine	Number of interventions (n = 65)
*Initial supervision*	
Single initial consultation	21
Frequent contacts during the initial period ^a^	3
Intensive training during the initial period ^b^	20
Not performed	21
*Strategies adopted during self-management*	
Periodic calls	30
Regular meetings	6
Sporadic supervised sessions	3
None	26

^a^ *Frequent contact during the initial period* included studies where authors repeatedly checked for patients’ adherence and comfort after the prescribed exercises via weekly in-person meetings, phone calls and emails.

^b^ *Intensive training during the initial period* involved studies in which authors provided a supervised period at the beginning of the program in order to educate patients and improve their long-term adherence.

With respect to the outcomes, many scales have been used across studies for CRF assessment, along with a set of secondary outcomes, as illustrated in Table 5. The most used scales were the Functional Assessment of Chronic Illness / Cancer Therapy–Fatigue scale (FACIT, 34% of the interventions), the Piper Fatigue Scale (PFS, 18%), the Brief Fatigue Inventory (11%), and the Multidimensional Fatigue Inventory (11%). As a complementary measure of CRF, some studies computed the maximal oxygen uptake (26%) and made cancer survivors perform functional tests for endurance and speed, such as the 6-min Walking Test (21%) and the 12-m Walking Test (8%).

**Table 5:** Primary outcome measures for cancer-related fatigue assessment.

Outcome measures	Number of interventions (n = 65)
Functional Assessment of Chronic Illness / Cancer Therapy–Fatigue (FACIT-F, FACT-F, FACT-ES, PROMIS-FACIT)	22
Piper fatigue Scale (PFS)	12
Brief Fatigue Inventory (BFI)	7
Multidimensional Fatigue Inventory (MFI)	7
Visual Analogue Scale (VAS), other linear analogue scales	5
Cancer Fatigue Scale (CFS)	3
Fatigue Symptom Inventory (FSI)	3
European Organization for Research and Treatment of Cancer QLQ-C30 (EORTC-QLQ-C30)	2
Multidimensional Fatigue Symptom Inventory (MFSI)	2
Fatigue Scale (FS)	1
Fatigue Severity Inventory (FSS)	1
MD Anderson Symptom Inventory (MDASI)	1
Profile of Mood States (POMS)	1
Schwartz Cancer Fatigue Scale (SCFS)	1

Note: Some studies used more than one CRF-related scale

The reported efficacy of the interventions provided in the included studies is illustrated in Table 6 and is grouped by study design. Among the RCTs, almost half of them (21/44) reported significant between-group differences after self-managed PA. We also mapped the results of the RCTs only by grouping them by initial supervision and strategies adopted to promote adherence to self-managed treatment (Table 7).

**Table 6:** Efficacy of self-managed PA protocols (smPA) in reducing CRF compared to the control treatment (when available) and the baseline values. Results are reported and split by study design. Both raw data and percentages computed over the total (n = 65) are presented.

Study results	Number of interventions, n = 65 (% of the studies over the total)
*Randomized controlled trial (n = 44)*	
Effective treatment *^,^^†^	21 (32%)
Effective treatment ^†^	11 (17%)
Ineffective treatment	12 (19%)
*Single-arm trial (n = 10)*	
Effective treatment ^†^	6 (9%)
Ineffective treatment	4 (6%)
*Pilot study (n = 9)*	
Effective treatment *^,^^†^	2 (3%)
Effective treatment ^†^	3 (5%)
Ineffective treatment	4 (6%)
*Non-randomized trial (n = 2)*	
Effective treatment *^,^^†^	1 (1.5%)
Ineffective treatment	1 (1.5%)

* Statistically significant between-groups difference (compared to control group): the effect of self-managed PA is greater than that of the control treatment;

^†^ statistically significant reduction of CRF over time in the intervention group (compared to baseline values): the effect of smPA is equal to that of the control treatment.

**Table 7:** RCT results based on the initial supervision provided before self-care and on strategies employed to promote adherence during self-managed activities. Counts are presented both as raw data and percentages computed over the total (n = 44).

Study results	Number of interventions in RCTs, n = 44 (% of the RCTs over the total)
***Initial supervision (n = 44)***	
*Single initial consultation (n = 14)*	
Effective treatment *^,^^†^	8 (18%)
Effective treatment ^†^	1 (2%)
Ineffective treatment	5 (11%)
*Frequent contacts during the initial period* ^a^ *(n = 1)*	
Effective treatment *^,^^†^	1 (2%)
*Intensive training during the initial period* ^b^ *(n = 16)*	
Effective treatment *^,^^†^	6 (14%)
Effective treatment ^†^	6 (14%)
Ineffective treatment	4 (9%)
*Not delivered (n = 13)*	
Effective treatment *^,^^†^	6 (14%)
Effective treatment ^†^	4 (9%)
Ineffective treatment	3 (7%)
***Strategy to increase adherence (n = 44)***	
*Periodic calls (n = 16)*	
Effective treatment *^,^^†^	8 (19%)
Effective treatment ^†^	3 (7%)
Ineffective treatment	5 (11%)
*Regular meetings (n = 6)*	
Effective treatment *^,^^†^	3 (7%)
Effective treatment ^†^	2 (5%)
Ineffective treatment	1 (2%)
*Sporadic supervised sessions (n = 3)*	
Effective treatment *^,^^†^	1 (2%)
Effective treatment ^†^	1 (2%)
Ineffective treatment	1 (2%)
*None (n = 19)*	
Effective treatment *^,^^†^	9 (21%)
Effective treatment ^†^	5 (11%)
Ineffective treatment	5 (11%)

* Statistically significant between-groups difference (compared to the control group): the effect of smPA is greater than that of the control treatment;

^†^ statistically significant within-group difference in the intervention group (compared to baseline values): the effect of smPA is equal to that of the control treatment.

^a^ *Frequent contact during the initial period* included studies where authors repeatedly checked for patients’ adherence and comfort after the prescribed exercises via weekly in-person meetings, phone calls and emails.

^b^ *Intensive training during the initial period* involved studies in which authors provided a supervised period at the beginning of the program in order to educate patients and improve their long-term adherence.

## Discussion

In this scoping review, we collected the available evidence regarding self-managed activities involving any type of PA for CS suffering from CRF. Sixty-five interventions were found: most of them involved CS enrolled in aerobic or resistance exercise programs, lasting 3–5 months and with at least 3 weekly sessions. Some programs included strategies to increase patients’ adherence and self-care inclusion in their daily routine, as with an initial supervised training phase or with periodic phone calls inquiring about patients’ comfort and needs. All details about treatments and results have been summarized in tables to facilitate the flow and the reading for clinicians for as a quick reference and for the benefit of clinicians interested in the topic.

Among the papers of interest to this scoping review, the terms “self-care” or “self-management” were found in only half of the studies when searching titles, keywords, and abstracts. This indicates that the term “self-management” is still not properly classified in the databases. Consequently, researchers might encounter some difficulties in retrieving studies by electronic searches. The in-depth hand search we performed, starting from the references of the already included studies, addressed this issue. Our scoping review synthesizes the available literature and can be used as a reference by researchers interested in this topic.

Scoping reviews are meant for map the literature around a broad-ranging topic and are useful for paving the way for future studies and highlighting any gaps that need to be filled [14]. The high prevalence of CRF even after the end of a cancer therapy [4] plus the increasing need to empower patients in the self-management of their chronic outcomes [10] explains the interest surrounding this topic, as demonstrated by the elevated number of studies included in this review that shows the growing number of targeted studies being published. Before 2010 only 11 studies had been published, while these increased to 39 papers during the last decade (2010–2019), and from 2020 on, there has been already 13 published papers.

In order to be considered eligible for the current review, studies had to have a professional prescribing some form of self-managed PA and, in the long run, these exercises had to be performed independently. Since CS are usually part of care programs, the prescriber is usually the health care professional responsible for the patient’s program. This figure may vary from country to country depending on how a national healthcare system is structured and on the roles assigned to each professional in the management of CS (e.g. oncologist, general practitioner, physiatrist, physiotherapist, occupational therapist). In order to ensure the inclusion of self-managed studies that could reflect a true self-management of CRF, we set a reasonable cutoff for the frequency of in-person meetings, which was at most every fortnight. In contrast, no limit was set for contacts over the phone or reminders sent by text message.

Most of the included studies are two-arms RCTs, comparing self-managed intervention with usual-care, which usually consisted in informing patients about the benefits of exercising without formally prescribing any specific activity (see Table 2). Breast cancer was the most studied type of tumor in the included papers (43 out of 65), probably because of its high incidence and survival rate. On the contrary, although prostate cancer is the most frequent type found in the male population, there is not a proportionately comparable number of studies for this population. Higher dropout rates and reduced participation in research studies have been reported in the male population, thus limiting the availability of results on this topic [83]. The findings in our review are consistent with the current literature and highlight a meaningful gap that needs to be addressed. On the one hand, the low male representation in studies included in future reviews may overall limit the efficacy of the results. On the other hand, the numerous benefits produced by PA in CS supports the need to find effective strategies to promote self-managed activities in the male population as well, first and foremost by raising awareness among healthcare providers, who are involved since the early stages of cancer therapy.

Several types of interventions have been found among studies, including aerobic or resistance exercises, programs combining both mind-body practices and programs with a multidimensional approach (see Table 3). Studies that provided mind-body practices were considered eligible since they involve the performance of gentle movements that promote the improvement of the physical component of CRF, even when combined with meditation which addresses the cognitive and emotional domains of the symptoms. This choice is in line with a previous review on the effectiveness of exercising, which included mind-body practices as eligible interventions [6]. When only aerobic exercise was suggested, the most frequent recommendation was simply to walk, according to the national CS guidelines [84]. Combining different types of activities involves more effort and some skill, especially for those exercises that strengthen various muscle groups, activities to improve balance, flexibility, and controlled breathing. Mind-body practices also incorporated a variety of activities, from meditative walking to the actual practice of Yoga, Tai Chi, and Qigong (see the S1 Table for a detailed summary of the interventions). This hurdle for CS, justifies the initial support provided by the professional, who gradually integrates the practice into the patient’s daily routine.

Among studies the duration of the self-managed interventions varied from 1–13 months (see Table 3). The majority of the studies (43%) included 3–5-month treatments, and only a few studies had follow-ups that lasted over a year. Thirty percent of the interventions were shorter, while only nine studies lasted less than 2 months. These results suggest that it is likely to take a reasonably long period of time in order to see any significant change in a syndrome as complex as CRF. The length of the intervention is key when designing future studies.

As demonstrated by our results, a common consensus regarding the frequency of a treatment does not seem to exist yet. Daily exercise varied between 1–2 times per week to every day. The low activity frequency described by six studies may be related to the fear of triggering the perception of fatigue, since a balance must be struck between symptom improvement, due to adequate training, and exacerbation, due to excessive exercise intensity [15]. However, 58% of the authors suggested exercising at least three times per week or 150 min/week, as per the National CS Guidelines [84], since frequent PA is necessary to counteract a condition as complex as CRF. Even, some of the most recent studies clearly stated that performing daily self-care practices is beneficial. These higher frequency doses are consistent with the goal of integrating PA as part of the patient’s daily routine. This would enable self-care to become an integral part of the survivors’ routine. Some authors have suggested that over time the perception of CRF may change due to a shift in the sensitivity threshold [40]. In this case, treatments should be tapered accordingly, with a gradual increase of the workload over time.

As often outlined in this review, self-managed programs may include initial training and strategies to foster long-term adherence. According to the focus of our review, when reporting and discussing the results, we only considered the duration of the self-managed period. The duration of the initial supervised period, if present, was reported in the S1 Table. Complex PA normally requires an initial period of training to help CS learn new exercises and activities. Hence, when designing new protocols, researchers should consider the duration of the initial supervision as a relevant parameter to increase the likelihood of long-term adhesion. Among the studies included in this review, the duration of this phase was on average 2 to 3 months (see S1 Table for raw data). Percentagewise, compared to the whole intervention period, this phase ranged between 3% and 58% mainly depending on the duration of the follow-up period.

During the self-managed PA period, about 60% of authors planned strategies to check on patient adherence. In 39 out of 65 interventions patient talked to a professional on an occasional basis, either in-person or over the phone. More specifically, mild remote supervision via phone calls may represent a strategy worth mentioning so as to help patients adhere to the program. This has several advantages: it requires little commitment in terms of economic and time resources for both patients and health professionals and patients do not feel left out. A satisfying level of treatment adherence is expected to result in a better outcome in terms of CRF relief [15]. However, this is not always evident when looking at the results (see Table 7), where studies were grouped based on the presence or absence of an occasional contact with the patients. It is also worth noting that adherence rate was not recorded in all the studies, while we think this parameter is key and should be recorded in future trials.

Our scoping review highlighted a wide range of professionals who designed and lead the self-managed pathways (see Table 3). These were either healthcare professionals, such as physiotherapists and nurses, or well-being professionals, e.g. mind-body teachers, or even exercise physiologists. In 17% of studies at least two professional figures were involved. This is in line with the Chronic Care Model [10], that supports the multidisciplinary collaboration between healthcare professionals as being essential to build a solid support network for all the issues CS can experience during their recovery. In the future, it will be increasingly important to establish collaborations between the healthcare professionals who follow the patient while still hospitalized and undergoing cancer therapies and other figures who can provide support through external activities that the patient can choose independently according to their preferences (gym sessions, groups of adapted PA, interactive online modules, peer support groups, psychological and nutritional support) and with the least possible burden to the national health systems. The Chronic Care Model shows clinicians how to build sustainable self-managed treatments, in which survivors develop self-efficacy skills, giving patients the best possible tools to make informed decisions regarding their health, contacting the health professional during appointments or when experiencing adverse effects [10]. Due to the requirement of keeping in touch with the health professionals, it should be reiterated that self-care cannot be a free-of-charge alternative [85]. To ensure that health systems begin to systematically introduce self-managed treatments within the services provided, new studies should highlight the favorable cost-effectiveness ratio of these methodologies compared to ending the treatment at discharge after a short period of only supervised sessions. This often leads to re-hospitalization and in the long term to increased costs for the national health systems [40, 86].

Among studies, the assessment of CFR was based on clinical scales, consisting of a set of items. The FACIT-F and the PFS were used in about half of the studies (see Table 5), while as many as 14 scales were found across all studies. These typically share common items, including CRF intensity, limitations in physical functioning, sense of tiredness, heaviness, and exhaustion, cognitive difficulties (lack of memory and concentration), and the ability to perform the activities of daily living. In general, the total score of these scales is obtained by summing up the single item scores, assessed on a Likert scale. Interestingly, some of these scales have already undergone a thorough analysis of their metric properties, according to the Rasch model [87–89] and have been upgraded from ordinal to numerical scales, thus being more suited for being used in future research studies.

We found that the efficacy of self-managed interventions reported by the individual studies varied substantially–see Tables 6 and 7. In line with the aim of a scoping review, we did not provide a statistical synthesis of treatment efficacy (effect size) across studies, weighted by each sample size, as in meta-analyses. Hence, no inference on the overall efficacy of self-managed PA can be done. Instead, our findings outline several factors to take into consideration when designing a meta-analysis, including the choice of the scales, frequency, duration, and type of intervention, the amount of supervision and adherence rate.

New clinical intervention studies on self-managed PA in CS can benefit from this scoping review including a detailed overview of what has already been done in literature. This is especially important since all the categories reported in column of the S1 Table (e.g. duration, frequency, type of supervision) must be taken into account when designing new clinical trials. Moreover, when designing new studies on the effect of self-care of CRF, secondary outcomes that provide information about fatigability and physical ability should also be considered, since they provide a more complete picture in terms of level of impairment during functional tests. This is in line with the result in this current review, where 74% of the studies combined at least a secondary outcome relative to physical performance (see S1 Table).

According to the Chronic Care Model [10] and the insights provided by some of the studies included in this review, we believe that an integrated approach is necessary for the management of a chronic issue such as the CRF in CS. An initial period of PA training may be appropriate to ease patients into these lifestyle changes. Also, self-care clinical protocols should always require periodic follow-ups to monitor patient engagement, support self-management practices, and monitor any changes that need immediate intervention, according to the risk stratification. Clinical trials should also be consistent with this vision, integrating self-managed PA within a stepwise continuum of care to ensure better rates of external validity in their results.

### Strengths and limitations

This is the first scoping review, which focuses on all self-managed PA when dealing with CRF in CS. The main strengths of this review are the robust literature search developed by a specialist at our Institution and the grouping of the characteristics included in all the studies in terms of treatment protocol (e.g. exercise, duration, frequency, supervision and effectiveness).

Among the papers of interest to this scoping review, the terms “self-care” or “self-management” were found in only half of the studies when searching titles, keywords, and abstracts. This may have limited the indexing of the studies within databases and the chance of retrieving them, so a comprehensive hand search was performed. However, we still could have missed some relevant papers, and this may be a limitation of our study.

Moreover, in this review we considered only interventions that involved at least one type of PA as their main focus. Other studies dealing with CRF management are also available in literature. However, they are more oriented towards the psychological and cognitive aspects of CRF, operating through cognitive-behavioral approaches, coping strategies, and educational approaches that increase self-efficacy skills [90–93]. Future specific reviews may provide adequate summaries of these interventions. Further investigations could also provide a synthesis of the results obtained combining different interventions (e.g. PA, psychological, nutritional), to understand which is the best combination to address CRF in CS.

## Conclusions

Exercising is a type of PA that is known to be beneficial in CRF. This review collected all available studies which focused on CS performing self-managed activities after an initial coaching and training by a professional. Our work provides useful insights for professionals working with cancer survivors who suffer from CRF empowering them by teaching self-managed approaches-techniques.

This scoping review also highlighted the main characteristics of a self-managed intervention that researchers should consider when designing either a new clinical trial or a systematic review to produce a quantitative synthesis with clinical and practical implications.

## Supporting information

S1 Table List of all studies included in the scoping review and their main characteristics. RCT Randomized Controlled Trial, PT physiotherapist; IG Intervention Group; CG Control Group; NA Not Applicable; ^†^ statistical significant difference within-group (compared to baseline values); * statistical significant difference between-groups (compared to control group).
(DOCX)

S2 Table PRISMA checklist. (DOCX)

## References

[1] National Cancer Institute. Cancer Statistics. 2020.

[2] Torre LA, Siegel RL, Ward EM, Jemal A. Global cancer incidence and mortality rates and trends—An update. Cancer Epidemiol Biomarkers Prev. 2016;25: 16–27. doi: 10.1158/1055-9965.EPI-15-0578 26667886

[3] Tralongo P, Carnio S, Giusti R, Pescarenico MG, Caraceni A, Pace A, et al. Linee guida Lungoviventi. Linee Guid AIOM. 2018; 1–61.

[4] Koornstra RHT, Peters M, Donofrio S, Van den Borne B, De Jong FA. Management of fatigue in patients with cancer—A practical overview. Cancer Treat Rev. 2014;40: 791–799. doi: 10.1016/j.ctrv.2014.01.004 24576643

[5] Berger AM, Mooney K, Banerjee C, Breitbart WS, Carpenter KM, Chang Y, et al. Cancer-Related Fatigue. Natl Compr Cancer Netw. 2020.

[6] Cramp F, Byron-Daniel J. Exercise for the management of cancer-related fatigue in adults (Review). Cochrane Database Syst Rev. 2012;2012. doi: 10.1002/14651858.CD006145.pub3 23152233 PMC8480137

[7] Mustian KM, Alfano CM, Heckler CE, Kleckner AS, Kleckner IR, Leach CR, et al. Comparison of Pharmaceutical, Psychological, and Exercise Treatments for Cancer-Related Fatigue: A Meta-Analysis. JAMA Oncol. 2017;4: 961–968. doi: 10.1001/jamaoncol.2016.6914 28253393 PMC5557289

[8] Tralongo P, Pescarenico AG, Surbone A, Bordonaro S, Berretta M, Di Mari A. Physical needs of long-term cancer patients. Anticancer Res. 2017;37: 4733–4746. doi: 10.21873/anticanres.11880 28870892

[9] Boland L, Bennett K, Connolly D. Self-management interventions for cancer survivors: a systematic review. Support Care Cancer. 2018;26: 1585–1595. doi: 10.1007/s00520-017-3999-7 29199362

[10] Wagner EH, Austin BT, Davis C, Hindmarsh M, Schaefer J, Bonomi A. Improving chronic illness care: Translating evidence into action. Health Aff. 2001;20: 64–78. doi: 10.1377/hlthaff.20.6.64 11816692

[11] Turner R, Steed L, Quirk H, Greasley R, Saxton J, Taylor S, et al. Interventions for promoting habitual exercise in people living with and beyond cancer. Cochrane Database Syst Rev. 2013. doi: 10.1002/14651858.CD010192.pub2 24065550

[12] Tricco AC, Lillie E, Zarin W, O’Brien KK, Colquhoun H, Levac D, et al. PRISMA extension for scoping reviews (PRISMA-ScR): Checklist and explanation. Ann Intern Med. 2018;169: 467–473. doi: 10.7326/M18-0850 30178033

[13] Peters MDJ, Godfrey CM, McInerney P, Munn Z, Tricco AC, Khalil H. Chapter 11: Scoping reviews. In: Aromataris E, Munn Z, editors. JBI Reviewer´s Manual. Joanna Briggs Institute Reviewer’s Manual, JBI, 2020; 2020. pp. 407–452. https://reviewersmanual.joannabriggs.org/

[14] Munn Z, Peters MDJ, Stern C, Tufanaru C, McArthur A, Aromataris E. Systematic review or scoping review? Guidance for authors when choosing between a systematic or scoping review approach. BMC Med Res Methodol. 2018;18: 143. doi: 10.1186/s12874-018-0611-x 30453902 PMC6245623

[15] Kessels E, Husson O, van der Feltz-Cornelis CM. The effect of exercise on cancer-related fatigue in cancer survivors: A systematic review and meta-analysis. Neuropsychiatr Dis Treat. 2018;14: 479–494. doi: 10.2147/NDT.S150464 29445285 PMC5810532

[16] Tian L, Lu HJ, Lin L, Hu Y. Effects of aerobic exercise on cancer-related fatigue: a meta-analysis of randomized controlled trials. Support Care Cancer. 2016;24: 969–983. doi: 10.1007/s00520-015-2953-9 26482381

[17] Gardner JR, Livingston PM, Fraser SF. Effects of exercise on treatment-related adverse effects for patients with prostate cancer receiving androgen-deprivation therapy: A systematic review. J Clin Oncol. 2014;32: 335–346. doi: 10.1200/JCO.2013.49.5523 24344218

[18] Page MJ, McKenzie JE, Bossuyt PM, Boutron I, Hoffmann TC, Mulrow CD, et al. The PRISMA 2020 statement: An updated guideline for reporting systematic reviews. BMJ. 2021;372. doi: 10.1136/bmj.n71 33782057 PMC8005924

[19] US National Coalition for Cancer Survivorship. Defining cancer survivorship. [cited 24 Mar 2022]. https://canceradvocacy.org/defining-cancer-survivorship/

[20] Baruth M, Wilcox S, Der Ananian C, Heiney S. Effects of Home-Based Walking on Quality of Life and Fatigue Outcomes in Early Stage Breast Cancer Survivors: A 12-Week Pilot Study. J Phys Act Health. 2015;12: S110–S118. doi: 10.1123/jpah.2012-0339 23963636

[21] Baumann FT, Bieck O, Oberste M, Kuhn R, Schmitt J, Wentrock S, et al. Sustainable impact of an individualized exercise program on physical activity level and fatigue syndrome on breast cancer patients in two German rehabilitation centers. Support Care Cancer. 2017;25: 1047–1054. doi: 10.1007/s00520-016-3490-x 27942857

[22] Broderick JM, Guinan E, Kennedy MJ, Hollywood D, Courneya KS, Culos-Reed SN, et al. Feasibility and efficacy of a supervised exercise intervention in de-conditioned cancer survivors during the early survivorship phase: The PEACH trial. J Cancer Surviv. 2013;7: 551–562. doi: 10.1007/s11764-013-0294-6 23749688

[23] Carter SJ, Hunter GR, McAuley E, Courneya KS, Anton PM, Rogers LQ. Lower rate-pressure product during submaximal walking: a link to fatigue improvement following a physical activity intervention among breast cancer survivors. J Cancer Surviv. 2016;10: 927–934. doi: 10.1007/s11764-016-0539-2 27061740 PMC5018414

[24] Chaoul A, Milbury K, Spelman A, Basen-Engquist K, Hall MH, Wei Q, et al. Randomized trial of Tibetan yoga in patients with breast cancer undergoing chemotherapy. Cancer. 2018;124: 36–45. doi: 10.1002/cncr.30938 28940301 PMC5735004

[25] Cornette T, Vincent F, Mandigout S, Antonini MT, Leobon S, Labrunie A, et al. Effects of home-based exercise training on VO2 in breast cancer patients under adjuvant or neoadjuvant chemotherapy (SAPA): A randomized controlled trial. Eur J Phys Rehabil Med. 2016;52: 223–232. 25986222

[26] Courneya KS, Friedenreich CM, Sela RA, Quinney HA, Rhodes RE, Handman M. The group psychotherapy and home-based physical exercise (GROUP-HOPE) trial in cancer survivors: Physical fitness and quality of life outcomes. Psychooncology. 2003;12: 357–374. doi: 10.1002/pon.658 12748973

[27] Damush TM, Perkins A, Miller K. The implementation of an oncologist referred, exercise self-management program for older breast cancer survivors. Psychooncology. 2006;15: 884–890. doi: 10.1002/pon.1020 16378317

[28] Delrieu L, Pialoux V, Pérol O, Morelle M, Martin A, Friedenreich C, et al. Feasibility and health benefits of an individualized physical activity intervention in women with metastatic breast cancer: Intervention study. JMIR mHealth uHealth. 2020;8. doi: 10.2196/12306 32012082 PMC7013652

[29] Dieli-Conwright CM, Courneya KS, Demark-Wahnefried W, Sami N, Lee K, Sweeney FC, et al. Aerobic and resistance exercise improves physical fitness, bone health, and quality of life in overweight and obese breast cancer survivors: A randomized controlled trial. Breast Cancer Res. 2018;20: 1–10. doi: 10.1186/s13058-018-1051-6 30340503 PMC6194749

[30] Donnelly CM, Blaney JM, Lowe-Strong A, Rankin JP, Campbell A, McCrum-Gardner E, et al. A randomised controlled trial testing the feasibility and efficacy of a physical activity behavioural change intervention in managing fatigue with gynaecological cancer survivors. Gynecol Oncol. 2011;122: 618–624. doi: 10.1016/j.ygyno.2011.05.029 21689848

[31] Galiano-Castillo N, Cantarero-Villanueva I, Fernández-Lao C, Ariza-García A, Díaz-Rodríguez L, Del-Moral-Ávila R, et al. Telehealth system: A randomized controlled trial evaluating the impact of an internet-based exercise intervention on quality of life, pain, muscle strength, and fatigue in breast cancer survivors. Cancer. 2016;122: 3166–3174. doi: 10.1002/cncr.30172 27332968

[32] Gokal K, Wallis D, Ahmed S, Boiangiu I, Kancherla K, Munir F. Effects of a self-managed home-based walking intervention on psychosocial health outcomes for breast cancer patients receiving chemotherapy: a randomised controlled trial. Support Care Cancer. 2016;24: 1139–1166. doi: 10.1007/s00520-015-2884-5 26275768

[33] Gracey JH, Watson M, Payne C, Rankin J, Dunwoody L. Translation research: “Back on Track”, a multiprofessional rehabilitation service for cancer-related fatigue. BMJ Support Palliat Care. 2016;6: 94–96. doi: 10.1136/bmjspcare-2014-000692 25526904 PMC4789756

[34] Grégoire C, Bragard I, Jerusalem G, Etienne AM, Coucke P, Dupuis G, et al. Group interventions to reduce emotional distress and fatigue in breast cancer patients: A 9-month follow-up pragmatic trial. Br J Cancer. 2017;117: 1442–1449. doi: 10.1038/bjc.2017.326 28926526 PMC5680472

[35] Hathiramani S, Pettengell R, Moir H, Younis A. Relaxation versus exercise for improved quality of life in lymphoma survivors—a randomised controlled trial. J Cancer Surviv. 2020. doi: 10.1007/s11764-020-00941-4 32986231 PMC7520510

[36] Hoffman AJ, Brintnall RA, Given BA, von Eye A, Jones LW, Brown JK. Using Perceived Self-efficacy to Improve Fatigue and Fatigability In Postsurgical Lung Cancer Patients. Cancer Nurs. 2017;40: 1–12. doi: 10.1097/NCC.0000000000000378 27135752 PMC5086324

[37] Huang HP, Wen FH, Yang TY, Lin YC, Tsai JC, Shun SC, et al. The effect of a 12-week home-based walking program on reducing fatigue in women with breast cancer undergoing chemotherapy: A randomized controlled study. Int J Nurs Stud. 2019;99: 103376. doi: 10.1016/j.ijnurstu.2019.06.007 31442785

[38] Husebø AML, Dyrstad SM, Mjaaland I, Søreide JA, Bru E. Effects of scheduled exercise on cancer-related fatigue in women with early breast cancer. Sci World J. 2014;2014. doi: 10.1155/2014/271828 24563628 PMC3915861

[39] Jacot W, Arnaud A, Jarlier M, Lefeuvre‐plesse C, Dalivoust P, Senesse P, et al. Brief hospital supervision of exercise and diet during adjuvant breast cancer therapy is not enough to relieve fatigue: A multicenter randomized controlled trial. Nutrients. 2020;12: 1–24. doi: 10.3390/nu12103081 33050321 PMC7600233

[40] Kampshoff CS, van Dongen JM, van Mechelen W, Schep G, Vreugdenhil A, Twisk JWR, et al. Long-term effectiveness and cost-effectiveness of high versus low-to-moderate intensity resistance and endurance exercise interventions among cancer survivors. J Cancer Surviv. 2018;12: 417–429. doi: 10.1007/s11764-018-0681-0 29497963 PMC5956032

[41] Kaur G, Prakash G, Malhotra P, Ghai S, Kaur S, Singh M, et al. Home-Based yoga program for the patients suffering from malignant lymphoma during chemotherapy: A feasibility study. Int J Yoga. 2018;11: 249. doi: 10.4103/ijoy.IJOY_17_18 30233121 PMC6134742

[42] Kim SSHS, Ko YH, Song Y, Kang MJ, Lee H, Kim SSHS, et al. Pre-post analysis of a social capital-based exercise adherence intervention for breast cancer survivors with moderate fatigue: a randomized controlled trial. Support Care Cancer. 2020;28: 5281–5289. doi: 10.1007/s00520-020-05363-7 32103358

[43] Koch AK, Rabsilber S, Lauche R, Kümmel S, Dobos G, Langhorst J, et al. The effects of yoga and self-esteem on menopausal symptoms and quality of life in breast cancer survivors—A secondary analysis of a randomized controlled trial. Maturitas. 2017;105: 95–99. doi: 10.1016/j.maturitas.2017.05.008 28551083

[44] Komatsu H, Yagasaki K, Yamauchi H, Yamauchi T, Takebayashi T. A self-directed home yoga programme for women with breast cancer during chemotherapy: A feasibility study. Int J Nurs Pract. 2016;22: 258–266. doi: 10.1111/ijn.12419 26643264 PMC5064641

[45] Koutoukidis DA, Land J, Hackshaw A, Heinrich M, McCourt O, Beeken RJ, et al. Fatigue, quality of life and physical fitness following an exercise intervention in multiple myeloma survivors (MASCOT): an exploratory randomised Phase 2 trial utilising a modified Zelen design. Br J Cancer. 2020;123: 187–195. doi: 10.1038/s41416-020-0866-y 32435057 PMC7374110

[46] Ligibel JA, Meyerhardt J, Pierce JP, Najita J, Shockro L, Campbell N, et al. Impact of a Telephone-Based Physical Activity Intervention upon Breast Cancer. Breast Cancer res Treat. 2012;132: 617–632. doi: 10.1007/s10549-011-1882-7.Impact PMC3353544 22113257

[47] Loh SY, Lee SY, Murray L. The Kuala Lumpur Qigong Trial for Women in the Cancer Survivorship Phase-Efficacy of a Three-Arm RCT to Improve QOL. Asian Pacific J Cancer Prev. 2014;15: 8127–8134. doi: 10.7314/APJCP.2014.15.19.8127 25338995

[48] Mazanec SR, Miano S, Baer L, Campagnaro EL, Sattar A, Daly BJ. A family-centered intervention for the transition to living with multiple myeloma as a chronic illness: A pilot study. Appl Nurs Res. 2017;35: 86–89. doi: 10.1016/j.apnr.2017.03.003 28532734

[49] Mijwel S, Jervaeus A, Bolam KA, Norrbom J, Bergh J, Rundqvist H, et al. High-intensity exercise during chemotherapy induces beneficial effects 12 months into breast cancer survivorship. J Cancer Surviv. 2019;13: 244–256. doi: 10.1007/s11764-019-00747-z 30912010 PMC6482129

[50] Mock V, Pickett M, Ropka ME, Lin EM, Stewart KJ, Rhodes VA, et al. Fatigue and quality of life outcomes of exercise during cancer treatment. Cancer Pract. 2001;9: 119–127. doi: 10.1046/j.1523-5394.2001.009003119.x 11879296

[51] Mock V, Frangakis C, Davidson NE, Ropka ME, Pickett M, Poniatowski B, et al. Exercise manages fatigue during breast cancer treatment: A randomized controlled trial. Psychooncology. 2005;14: 464–477. doi: 10.1002/pon.863 15484202

[52] Moonsammy SH, Guglietti CL, Mina DS, Ferguson S, Kuk JL, Urowitz S, et al. A pilot study of an exercise & cognitive behavioral therapy intervention for epithelial ovarian cancer patients. J Ovarian Res. 2013;6: 1. doi: 10.1186/1757-2215-6-21 23557323 PMC3623735

[53] Mustian KM, Peppone L, Darling T V, Palesh O, Heckler CE, Morrow GR. A 4-week home-based aerobic and resistance exercise program during radiation therapy: a pilot randomized clinical trial. J Support Oncol. 2013;7: 158–67. Available: http://www.ncbi.nlm.nih.gov/pubmed/19831159 PMC3034389 19831159

[54] Nyrop KA, Callahan LF, Cleveland RJ, Arbeeva LL, Hackney BS, Muss HB. Randomized Controlled Trial of a Home‐Based Walking Program to Reduce Moderate to Severe Aromatase Inhibitor‐Associated Arthralgia in Breast Cancer Survivors. Oncologist. 2017;22: 1238–1249. doi: 10.1634/theoncologist.2017-0174 28698390 PMC5634775

[55] Ochi E, Tsuji K, Narisawa T, Shimizu Y, Kuchiba A, Suto A, et al. Cardiorespiratory fitness in breast cancer survivors: a randomised controlled trial of home-based smartphone supported high intensity interval training. BMJ Support Palliat Care. 2022;12: 33–37. doi: 10.1136/bmjspcare-2021-003141 34389552 PMC8862092

[56] Oldervoll LM, Kaasa S, Knobel H, Loge JH. Exercise reduces fatigue in chronic fatigued Hodgkins disease survivors—Results from a pilot study. Eur J Cancer. 2003;39: 57–63. doi: 10.1016/s0959-8049(02)00483-5 12504659

[57] Payne JK, Held J, Thorpe J, Shaw H. Effect of exercise on biomarkers, fatigue, sleep disturbances, and depressive symptoms in older women with breast cancer receiving hormonal therapy. Oncol Nurs Forum. 2008;35: 635–642. doi: 10.1188/08.ONF.635-642 18591167

[58] Pinto BM, Rabin C, Papandonatos GD, Frierson GM, Trunzo JJ, Marcus BH. Maintenance of effects of a home-based physical activity program among breast cancer survivors. Support Care Cancer. 2008;16: 1279–1289. doi: 10.1007/s00520-008-0434-0 18414905 PMC2747581

[59] Pinto BM, Papandonatos GD, Goldstein MG, Marcus BH, Farrell N. Home-based physical activity intervention for colorectal cancer survivors. Psychooncology. 2013;22: 54–64. doi: 10.1002/pon.2047 21905158

[60] Porter LS, Carson JW, Olsen M, Carson KM, Sanders L, Jones L, et al. Feasibility of a mindful yoga program for women with metastatic breast cancer: results of a randomized pilot study. Support Care Cancer. 2019;27: 4307–4316. doi: 10.1007/s00520-019-04710-7 30877596 PMC6745290

[61] Prieto-Gómez V, Yuste-Sánchez MJ, Bailón-Cerezo J, Romay-Barrero H, de la Rosa-Díaz I, Lirio-Romero C, et al. Effectiveness of Therapeutic Exercise and Patient Education on Cancer-Related Fatigue in Breast Cancer Survivors: A Randomised, Single-Blind, Controlled Trial with a 6-Month Follow-Up. J Clin Med. 2022;11. doi: 10.3390/jcm11010269 35012011 PMC8746078

[62] Qiao Y, van Londen GJ, Brufsky JW, Poppenberg JT, Cohen RW, Boudreau RM, et al. Perceived physical fatigability improves after an exercise intervention among breast cancer survivors: a randomized clinical trial. Breast Cancer. 2022;29: 30–37. doi: 10.1007/s12282-021-01278-1 34328623

[63] Rogers LQ, Fogleman A, Trammell R, Hopkins-Price P, Vicari S, Rao K, et al. Effects of a Physical Activity Behavior Change Intervention on Inflammation and Related Health Outcomes in Breast Cancer Survivors. Integr Cancer Ther. 2013;12: 323–335. doi: 10.1177/1534735412449687 22831916 PMC3909487

[64] Schröder ML, Stöckigt B, Binting S, Tissen-Diabaté T, Bangemann N, Goerling U, et al. Feasibility and Possible Effects of Mindful Walking and Moderate Walking in Breast Cancer Survivors: A Randomized Controlled Pilot Study With a Nested Qualitative Study Part. Integr Cancer Ther. 2022;21: 7–9. doi: 10.1177/15347354211066067 35045736 PMC8777370

[65] Schwartz AL. Daily fatigue patterns and effect of exercise in women with breast cancer. Cancer Pract. 2000;8: 16–24. doi: 10.1046/j.1523-5394.2000.81003.x 10732535

[66] Schwartz AL, Mori M, Gao R, Nail LM, King ME. Exercise reduces daily fatigue in women with breast cancer receiving chemotherapy. Med Sci Sports Exerc. 2001;33: 718–23. doi: 10.1097/00005768-200105000-00006 11323538

[67] Sheehan P, Denieffe S, Murphy NM, Harrison M. Exercise is more effective than health education in reducing fatigue in fatigued cancer survivors. Support Care Cancer. 2020;28: 4953–4962. doi: 10.1007/s00520-020-05328-w 32020356

[68] Spector D, Deal AM, Amos KD, Yang H, Battaglini CL. A pilot study of a home-based motivational exercise program for African American breast cancer survivors: Clinical and quality-of-life outcomes. Integr Cancer Ther. 2014;13: 121–132. doi: 10.1177/1534735413503546 24105359 PMC10568972

[69] Stan DL, Croghan KA, Croghan IT, Jenkins SM, Sutherland SJ, Cheville AL, et al. Randomized pilot trial of yoga versus strengthening exercises in breast cancer survivors with cancer-related fatigue. Support Care Cancer. 2016;24: 4005–4015. doi: 10.1007/s00520-016-3233-z 27129840

[70] van de Wiel HJ, Stuiver MM, May AM, van Grinsven S, Aaronson NK, Oldenburg HSA, et al. Effects of and lessons learned from an internet-based physical activity support program (With and without physiotherapist telephone counselling) on physical activity levels of breast and prostate cancer survivors: The pablo randomized controlled trial. Cancers (Basel). 2021;13. doi: 10.3390/cancers13153665 34359567 PMC8345041

[71] Van Waart H, Stuiver MM, Van Harten WH, Geleijn E, Kieffer JM, Buffart LM, et al. Effect of low-intensity physical activity and moderate- to high-intensity physical exercise during adjuvant chemotherapy on physical fitness, fatigue, and chemotherapy completion rates: Results of the PACES randomized clinical trial. J Clin Oncol. 2015;33: 1918–1927. doi: 10.1200/JCO.2014.59.1081 25918291

[72] VanderWalde NA, Martin MY, Kocak M, Morningstar C, Deal AM, Nyrop KA, et al. Randomized phase II study of a home-based walking intervention for radiation-related fatigue among older patients with breast cancer. J Geriatr Oncol. 2021;12: 227–234. doi: 10.1016/j.jgo.2020.09.013 32978102

[73] Vincent F, Labourey JL, Leobon S, Antonini MT, Lavau-Denes S, Tubiana-Mathieu N. Effects of a home-based walking training program on cardiorespiratory fitness in breast cancer patients receiving adjuvant chemotherapy: A pilot study. Eur J Phys Rehabil Med. 2013;49: 319–329. 23480974

[74] Vincent F, Deluche E, Bonis J, Leobon S, Antonini MT, Laval C, et al. Home-Based Physical Activity in Patients With Breast Cancer: During and/or After Chemotherapy? Impact on Cardiorespiratory Fitness. A 3-Arm Randomized Controlled Trial (APAC). Integr Cancer Ther. 2020;19. doi: 10.1177/1534735420969818 33228382 PMC7691904

[75] Wangnum K, Thanarojanawanich T, Chinwatanachai K, Jamprasert L, Maleehuan O, Janthakun V. Impact of the multidisciplinary education program in self-care on fatigue in lung cancer patients receiving chemotherapy. J Med Assoc Thai. 2013;96: 1601–1608. 24511726

[76] Wilson RW, Jacobsen PB, Fields KK. Pilot study of a home-based aerobic exercise program for sedentary cancer survivors treated with hematopoietic stem cell transplantation. Bone Marrow Transplant. 2005;35: 721–727. doi: 10.1038/sj.bmt.1704815 15696182

[77] Winters-Stone KM, Moe EL, Perry CK, Medysky M, Pommier R, Vetto J, et al. Enhancing an oncologist’s recommendation to exercise to manage fatigue levels in breast cancer patients: a randomized controlled trial. Support Care Cancer. 2018;26: 905–912. doi: 10.1007/s00520-017-3909-z 28965138

[78] Yang CY, Tsai JC, Huang YC, Lin CC. Effects of a home-based walking program on perceived symptom and mood status in postoperative breast cancer women receiving adjuvant chemotherapy. J Adv Nurs. 2011;67: 158–168. doi: 10.1111/j.1365-2648.2010.05492.x 20973811

[79] Young Kim J, Kyung Lee M, Hoon Lee D, Woo Kang D, Hee Min J, Won Lee J, et al. Effects of a 12-week home-based exercise program on quality of life, psychological health, and the level of physical activity in colorectal cancer survivors: a randomized controlled trial. Support Care Cancer. 2019;27: 2933–2940. doi: 10.1007/s00520-018-4588-0 30564936

[80] Yuen HK, Sword D. Home-based exercise to alleviate fatigue and improve functional capacity among breast cancer survivors. J Allied Health. 2007;36: 257–275. 19759996

[81] Yun YH, Lee KS, Kim YW, Park SY, Lee ES, Noh DY, et al. Web-based tailored education program for disease-free cancer survivors with cancer-related fatigue: A randomized controlled trial. J Clin Oncol. 2012;30: 1296–1303. doi: 10.1200/JCO.2011.37.2979 22412149

[82] Zhou Y, Cartmel B, Gottlieb L, Ercolano EA, Li F, Harrigan M, et al. Randomized Trial of Exercise on Quality of Life in Women With Ovarian Cancer: Women’s Activity and Lifestyle Study in Connecticut (WALC). J Natl Cancer Inst. 2017;109: 1–7. doi: 10.1093/jnci/djx072 30053074 PMC6515522

[83] Ford CG, Vowles KE, Smith BW, Kinney AY. Mindfulness and Meditative Movement Interventions for Men Living With Cancer: A Meta-analysis. Ann Behav Med. 2020; 1–14. doi: 10.1093/abm/kaz053 31773148 PMC7168578

[84] Schmitz KH, Courneya KS, Matthews C, Demark-Wahnefried W, Galvão DA, Pinto BM, et al. American college of sports medicine roundtable on exercise guidelines for cancer survivors. Med Sci Sports Exerc. 2010;42: 1409–1426. doi: 10.1249/MSS.0b013e3181e0c112 20559064

[85] Todd M. Self-management of chronic oedema in the community. Br J Community Nurs. 2014;19. doi: 10.12968/bjcn.2014.19.Sup4.S30 24704752

[86] van Waart H, van Dongen JM, van Harten WH, Stuiver MM, Huijsmans R, Hellendoorn-van Vreeswijk JAJH, et al. Cost–utility and cost-effectiveness of physical exercise during adjuvant chemotherapy. Eur J Heal Econ. 2018;19: 893–904. doi: 10.1007/s10198-017-0936-0 29086085

[87] Lerdal A, Kottorp A, Gay C, Aouizerat BE, Lee KA, Miaskowski C. A Rasch Analysis of Assessments of Morning and Evening Fatigue in Oncology Patients Using the Lee Fatigue Scale. J Pain Symptom Manage. 2016;51: 1002–1012. doi: 10.1016/j.jpainsymman.2015.12.331 26975624 PMC4902715

[88] Rowen D, Brazier J, Young T, Gaugris S, Craig BM, King MT, et al. Deriving a Preference-Based Measure for Cancer Using the EORTC QLQ-C30. Value Heal. 2011;14: 721–731. doi: 10.1016/j.jval.2011.01.004 21839411 PMC3811066

[89] Lundgren-Nilsson Å, Dencker A, Jakobsson S, Taft C, Tennant A. Construct validity of the Swedish version of the revised piper fatigue scale in an oncology sample—A rasch analysis. Value Heal. 2014;17: 360–363. doi: 10.1016/j.jval.2014.02.010 24968995

[90] Sandler CX, Goldstein D, Horsfield S, Bennett BK, Friedlander M, Bastick PA, et al. Randomized Evaluation of Cognitive-Behavioral Therapy and Graded Exercise Therapy for Post-Cancer Fatigue. J Pain Symptom Manage. 2017;54: 74–84. doi: 10.1016/j.jpainsymman.2017.03.015 28502786

[91] Børøsund E, Varsi C, Clark MM, Ehlers SL, Andrykowski MA, Sleveland HRS, et al. Pilot testing an app-based stress management intervention for cancer survivors. Transl Behav Med. 2020;10: 770–780. doi: 10.1093/tbm/ibz062 31330023 PMC7413188

[92] Coolbrandt A, Wildiers H, Laenen A, Aertgeerts B, De Casterlé BD, Van Achterberg T, et al. A nursing intervention for reducing symptom burden during chemotherapy. Oncol Nurs Forum. 2018;45: 115–128. doi: 10.1188/18.ONF.115-128 29251287

[93] Courtier N, Armes J, Smith A, Radley L, Hopkinson JB. Targeted self-management limits fatigue for women undergoing radiotherapy for early breast cancer: results from the ACTIVE randomised feasibility trial. Support Care Cancer. 2022;30: 389–400. doi: 10.1007/s00520-021-06360-0 34297221 PMC8298947

