# Supplementary material for: Self-managed physical activity in cancer survivors for the management of cancer-related fatigue: A scoping review
Source: PLoS One. 2022 Dec 21;17(12):e0279375. doi: 10.1371/journal.pone.0279375 (PMC9770433; doi:10.1371/journal.pone.0279375)
Supplement: S1 Table — RCT Randomized Controlled Trial, PT physiotherapist; IG Intervention Group; CG Control Group; NA Not Applicable; † statistical significant difference within-group (compared to baseline values); * statistical significant difference between-groups (compared to control group). (DOCX) [file pone.0279375.s001.docx]

**S1 Table.** **List of all studies included in the scoping review and their main characteristics.**

| **Author and Year** | **Study design** | **Cancer type and sample size** | **Health professional leading the study** | **Intervention** | **Strategy to maintain adherence** | **Recommended frequency** | **Duration** | **Initial supervision** | **Duration of the supervision in weeks (% of the supervision over the whole intervention)** | **Control group** | **Fatigue-related outcome measures** | **Complementary outcome measures** | **Follow up** | **Study results** |
| --- | --- | --- | --- | --- | --- | --- | --- | --- | --- | --- | --- | --- | --- | --- |
| Baruth et al. 2015 | Pilot study | Breast cancer, 32 participants | Other | Multidimensional program (physical, nutritional, psychological components) | None | ≥ 3 times/week | 3-5 months | Single initial consultation | NA | Usual care | FACT-F | CHAMPS, accelerometer, log | Not considered | Effective treatment for CRF^†*^ |
| Baumann et al. 2017 | RCT | Breast cancer, 194 patients | PT | Aerobic exercise program | Sporadic supervised sessions | Not indicated | ≥ 1 year | Intensive training during initial period | 3 (3%) | Usual care | MFI | No further outcomes | Not considered | Effective treatment for CRF^†*^ |
| Broderick et al. 2013 | RCT | Various, 43 patients | PT | Aerobic exercise program | None | ≥ 3 times/week | 3-5 months | Intensive training during initial period | 8 (40%) | Usual care | FACIT-F | VO2 max | Not considered | Effective treatment for CRF^†*^ |
| Carter et al. 2016 | RCT | Breast cancer, 152 patients | Exercise expert | Aerobic exercise program | Regular meetings | ≥ 3 times/week | 1-2 months | Intensive training during initial period | 6 (50%) | Usual care | 5-point linear scale | VO2 max | Not considered | Effective treatment for CRF^†*^ |
| Chaoul et al. 2018 | RCT | Breast cancer, 227 patients | Multidisciplinary team | Mind-body practice (yoga, tai chi, qigong...) | Regular meetings | Daily | ≥ 1 year | Intensive training during initial period | Unclear | Active control group / usual care | BFI | No further outcomes | Not considered | Effective treatment for CRF^†^ |
| Cornette et al. 2016 | RCT | Breast cancer, 42 participants | Exercise expert | Combined exercise program (aerobic, resistance, flexibility, stretching, balance…) | Periodic calls | 1-2 times/week | 6-11 months | Single initial consultation | NA | Usual care | MFI | VO2, 6MWT | 27 weeks (week 54) | Ineffective treatment for CRF |
| Courneya et al. 2003 | RCT | Various, 108 participants | Multidisciplinary team | Multidimensional program (physical, nutritional, psychological components) | None | Not indicated | 1-2 months | Single initial consultation | NA | Educational group | FACT-F | VO2 max | Not considered | Effective treatment for CRF^†*^ |
| Damush et al. 2006 | Single-arm trial | Breast cancer, 34 participants | Not specified | Combined exercise program (aerobic, resistance, flexibility, stretching, balance…) | Periodic calls | ≥ 3 times/week | 6-11 months | Frequent contacts during the initial period | NA | Not included | FACT-F | CHAMPS, Senior fitness test, accelerometer | Not considered | Effective treatment for CRF^†^ |
| Delrieu et al. 2020 | Single-arm trial | Breast cancer, 49 patients | Exercise expert | Aerobic exercise program | Periodic calls | Daily | 6-11 months | Not delivered | NA | Not included | PFS | EORTC-QLQ-C30, 6MWT, VO2 max | Not considered | Ineffective treatment for CRF |
| Dieli-Conwright et al. 2018 | RCT | Breast cancer, 100 participants | Exercise expert | Combined exercise program (aerobic, resistance, flexibility, stretching, balance…) | None | ≥ 3 times/week | 3-5 months | Intensive training during initial period | 16 (58%) | Usual care | BFI | VO2 max | Not considered | Effective treatment for CRF^†*^ |
| Donnelly et al. 2011 | RCT | Gynecological cancers, 33 participants | PT | Combined exercise program (aerobic, resistance, flexibility, stretching, balance…) | Periodic calls | ≥ 3 times/week | 3-5 months | Single initial consultation | NA | Usual care | MFSI-SF, FACIT-FS | 12mWT | 6 months | Effective treatment for CRF^†*^ |
| Galiano-Castillo et al. 2016 | RCT | Breast cancer, 81 participants | Not specified | Combined exercise program (aerobic, resistance, flexibility, stretching, balance…) | Periodic calls | ≥ 3 times/week | 1-2 months | Single initial consultation | NA | Usual care | PFS | No further outcomes | 6 months | Effective treatment for CRF^†*^ |
| Gokal et al. 2016 | RCT | Breast cancer, 50 patients | Not specified | Aerobic exercise program | None | ≥ 3 times/week | 3-5 months | Not delivered | NA | Usual care | FACT-F | Logs, pedometer | Not considered | Effective treatment for CRF^†*^ |
| Gracey et al. 2016 | Single-arm trial | Various, 17 participants | Not specified | Combined exercise program (aerobic, resistance, flexibility, stretching, balance…) | Periodic calls | ≥ 3 times/week | 1-2 months | Frequent contacts during the initial period | NA | Not included | FACIT-FS | ESAS, 6MWT, log, pedometer | Not considered | Effective treatment for CRF^†^ |
| Gregoire et al. 2017 | Non-randomized clinical trial | Breast cancer, 123 patients | Mind-body teacher | Mind-body practice (yoga, tai chi, qigong...) | None | Not indicated | 6-11 months | Intensive training during initial period | 6 (17%) | Educational group / usual care | EORTC-QLQ-C30 | No further outcomes | Not provided | Ineffective treatment for CRF |
| Hathiramani et al. 2020 | RCT | Lymphoma, 46 participants | Not specified | Combined exercise program (aerobic, resistance, flexibility, stretching, balance…) | None | ≥ 3 times/week | 3-5 months | Not delivered | NA | Active control group | EORTC-QLQ-C30 | No further outcomes | Not considered | Effective treatment for CRF^†*^ |
| Hoffman et al. 2017 | Pilot study | Lung cancer, 72 participants | Nurse | Combined exercise program (aerobic, resistance, flexibility, stretching, balance…) | Periodic calls | ≥ 3 times/week | 1-2 months | Single initial consultation | NA | Usual care | BFI | PSEFSM, 6MWT, SF-36, Borg scale, log | Not considered | Effective treatment for CRF^†*^ |
| Huang et al. 2019 | RCT | Breast cancer, 159 patients | Exercise expert | Aerobic exercise program | Periodic calls | ≥ 3 times/week | 3-5 months | Single initial consultation | NA | Usual care | BFI | 6MWT, KPS | Not considered | Effective treatment for CRF^†*^ |
| Husebø et al. 2014 | RCT | Breast cancer, 67 patients | Not specified | Combined exercise program (aerobic, resistance, flexibility, stretching, balance…) | Periodic calls | Daily | 6-11 months | Not delivered | NA | Usual care | SCFS-6 | 6MWT | Not provided | Ineffective treatment for CRF |
| Jacot et al. 2020 | RCT | Breast cancer, 360 participants | Multidisciplinary team | Combined exercise program (aerobic, resistance, flexibility, stretching, balance…) | None | 1-2 times/week | 6-11 months | Intensive training during initial period | 26 (52%) | Usual care | MFI | Sit-to-stand test | Not considered | Ineffective treatment for CRF |
| Kampshoff et al. 2018 | RCT | Various, 277 participants | PT | Combined exercise program (aerobic, resistance, flexibility, stretching, balance…) | Sporadic supervised sessions | Not indicated | ≥ 1 year | Intensive training during initial period | 12 (18%) | Active control group | MFI | VO2, accelerometer | Not considered | Ineffective treatment for CRF |
| Kaur et al. 2018 | Single-arm trial | Lymphoma, 14 participants | Mind-body teacher | Mind-body practice (yoga, tai chi, qigong...) | None | Daily | 1-2 months | Intensive training during initial period | Unclear | Not included | FACIT-F | No further outcomes | Not considered | Effective treatment for CRF^†^ |
| Kim et al. 2020 | RCT | Breast cancer, 48 participants | Exercise expert | Multidimensional program (physical, nutritional, psychological components) | None | 1-2 times/week | 1-2 months | Intensive training during initial period | 6 (50%) | Usual care | PFS | Korean-revised Global Physical Activity Questionnaire | Not considered | Effective treatment for CRF^†^ |
| Koch et al. 2017 | RCT | Breast cancer, 40 patients | Mind-body teacher | Mind-body practice (yoga, tai chi, qigong...) | None | Daily | 3-5 months | Intensive training during initial period | 12 (50%) | Usual care | FACIT-F | No further outcomes | Not provided | Ineffective treatment for CRF |
| Komatsu et al. 2016 | Single-arm trial | Breast cancer, 18 participants | Mind-body teacher | Mind-body practice (yoga, tai chi, qigong...) | None | Not indicated | 1-2 months | Single initial consultation | NA | Not included | CFS | No further outcomes | Not considered | Ineffective treatment for CRF |
| Koutoukidis et al. 2020 | RCT | Myeloma, 131 participants | PT | Combined exercise program (aerobic, resistance, flexibility, stretching, balance…) | Sporadic supervised sessions | ≥ 3 times/week | 3-5 months | Intensive training during initial period | 12 (50%) | Usual care | FACIT-F | VO2 max | 6 months | Effective treatment for CRF^†^ |
| Ligibel et al. 2012 | RCT | Breast and colorectal cancers, 121 participants | Other | Aerobic exercise program | Periodic calls | ≥ 3 times/week | 3-5 months | Not delivered | NA | Usual care | FACIT-FS | 6MWT, PASE-Q, accelerometer | Not considered | Effective treatment for CRF^†^ |
| Loh et al. 2014 | RCT | Breast cancer, 197 participants | Mind-body teacher | Mind-body practice (yoga, tai chi, qigong...) | None | Not indicated | 6-11 months | Intensive training during initial period | 8 (15%) | Active control group | FACIT-F | log | Not considered | Effective treatment for CRF^†^ |
| Mazanec et al. 2017 | Pilot study | Myeloma, 15 patients | Nurse | Aerobic exercise program | Periodic calls | ≥ 3 times/week | 3-5 months | Single initial consultation | NA | Usual care | PROMIS-fatigue | No further outcomes | Not considered | Effective treatment for CRF^†^ |
| Mijwel et al. 2019 | RCT | Breast cancer, 240 patients | Multidisciplinary team | Aerobic exercise program | Regular meetings | Not indicated | 6-11 months | Intensive training during initial period | 16 (30%) | Usual care | PFS | VO2 max | Not considered | Effective treatment for CRF^†*^ |
| same as above | RCT | Breast cancer, 240 patients | Multidisciplinary team | Combined exercise program (aerobic, resistance, flexibility, stretching, balance…) | Regular meetings | Not indicated | 6-11 months | Intensive training during initial period | 16 (30%) | Usual care | PFS | VO2 max | Not considered | Effective treatment for CRF^†*^ |
| Mock et al. 2001 | RCT | Breast cancer, 52 patients | Not specified | Aerobic exercise program | Periodic calls | 1-2 times/week | 3-5 months | Single initial consultation | NA | Usual care | PFS | 12mWT | Not considered | Effective treatment for CRF^†*^ |
| Mock et al. 2005 | RCT | Breast cancer, 119 patients | Nurse | Aerobic exercise program | Periodic calls | ≥ 3 times/week | 1-2 months | Single initial consultation | NA | Usual care | PFS | 12mWT | Not considered | Effective treatment for CRF^†*^ |
| Moonsammy et al. 2013 | Pilot study | Ovarian cancer, 19 participants | Multidisciplinary team | Combined exercise program (aerobic, resistance, flexibility, stretching, balance…) | Periodic calls | ≥ 3 times/week | 6-11 months | Single initial consultation | NA | Usual care | FACT-F | VO2 max | Not considered | Ineffective treatment for CRF |
| Mustian et al. 2008 | RCT | Breast and prostate cancer, 38 patients | Exercise expert | Combined exercise program (aerobic, resistance, flexibility, stretching, balance…) | None | Daily | 1-2 months | Single initial consultation | NA | Usual care | BFI | FACIT, 6MWT | Not considered | Effective treatment for CRF^†*^ |
| Nyrop et al. 2017 | RCT | Breast cancer, 62 participants | Not specified | Aerobic exercise program | None | ≥ 3 times/week | 1-2 months | Not delivered | NA | Usual care | VAS | Log, questionnaire | Not considered | Ineffective treatment for CRF |
| Ochi et al. 2021 | RCT | Breast cancer, 50 patients | Not specified | Aerobic exercise program | Periodic calls | ≥ 3 times/week | 3-5 months | Not delivered | NA | Usual care | Cancer Fatigue Scale | VO2 max, 6MWT | Not included | Effective treatment for CRF^†^ |
| Oldervoll et al. 2003 | Pilot study | Hodgkins lymphoma, 9 participants | Exercise expert | Aerobic exercise program | Periodic calls | ≥ 3 times/week | 3-5 months | Single initial consultation | NA | Usual care | Norvegian version of the Fatigue Questionnaire | VO2 max | Not considered | Effective treatment for CRF^†^ |
| Payne et al. 2008 | Pilot study | Breast cancer, 20 patients | Nurse | Aerobic exercise program | None | ≥ 3 times/week | 3-5 months | Not delivered | NA | Usual care | PFS | No further outcomes | Not considered | Ineffective treatment for CRF |
| Pinto et al. 2008 | RCT | Breast cancer, 86 patients | Not specified | Aerobic exercise program | Periodic calls | Not indicated | 6-11 months | Frequent contacts during the initial period | NA | Usual care | Linear analogue scale for fatigue | Rockport one-mile walk test | Not considered | Effective treatment for CRF^†*^ |
| Pinto et al. 2013 | RCT | Colorectal cancer, 46 participants | Not specified | Aerobic exercise program | Periodic calls | ≥ 3 times/week | 3-5 months | Not delivered | NA | Usual care | FACT-F | CHAMPS, accelerometer, VO2 max | 6, 12 months | Ineffective treatment for CRF |
| Porter et al. 2019 | Pilot study | Breast cancer, 63 patients | Mind-body teacher | Mind-body practice (yoga, tai chi, qigong...) | None | Daily | 3-5 months | Intensive training during initial period | 8 (40%) | Educational group | BFI | 6MWT | 3 months | Ineffective treatment for CRF |
| Prieto-Gomez et al. 2022 | RCT | Breast cancer, 80 patients | PT | Multidimensional program (physical, nutritional, psychological components) | None | ≥ 3 times/week | 1-2 months | Single initial consultation | NA | Similar activity, supervised | FACIT-F | 6MWT | 1, 4 months | Ineffective treatment for CRF |
| Qiao et al. 2022 | RCT | Breast cancer, 60 patients | Exercise expert | Aerobic exercise program | Regular meetings | ≥ 3 times/week | 3-5 months | Single initial consultation | NA | Usual care | FACT-ES | Pittsburgh Fatigability Scale | Not considered | Effective treatment for CRF^†^ |
| Rogers et al. 2013 | RCT | Breast cancer, 28 patients | Multidisciplinary team | Multidimensional program (physical, nutritional, psychological components) | Regular meetings | ≥ 3 times/week | 1-2 months | Intensive training during initial period | 6 (50%) | Usual care | FSI | No further outcomes | Not considered | Ineffective treatment for CRF |
| Schröder et al. 2022 | RCT | Breast cancer, 51 patients | Multidisciplinary team | Mind-body practice (yoga, tai chi, qigong...) | None | Not indicated | 1-2 months | Intensive training during initial period | 8 (50%) | Active control group | Cancer Fatigue Scale | No further outcomes | Not included | Effective treatment for CRF^†^ |
| Schwartz 2000 | Single-arm trial | Breast cancer, 31 patients | Not specified | Aerobic exercise program | None | ≥ 3 times/week | 1-2 months | Not delivered | NA | Not included | Linear analogue scale for CRF | 12mWT | Not considered | Effective treatment for CRF^†^ |
| Schwartz et al. 2001 | Single-arm trial | Breast cancer, 72 participants | Not specified | Aerobic exercise program | Periodic calls | ≥ 3 times/week | 1-2 months | Not delivered | NA | Not included | Linear analogue scale for CRF | 12mWT | Not considered | Effective treatment for CRF^†^ |
| Sheehan et al. 2020 | Non-randomized clinical trial | Various, 37 participants | Not specified | Aerobic exercise program | Periodic calls | 1-2 times/week | 1-2 months | Intensive training during initial period | 10 (38%) | Educational group | FACT-F | Log, 6MWT, IPAQ, C-reactive protein | At the end of the 26 weeks | Effective treatment for CRF^†*^ |
| Spector et al. 2014 | Single-arm trial | Breast cancer, 17 patients | Exercise expert | Combined exercise program (aerobic, resistance, flexibility, stretching, balance…) | Periodic calls | ≥ 3 times/week | 3-5 months | Single initial consultation | NA | Not included | FACIT-F | VO2 max | Not considered | Ineffective treatment for CRF |
| Stan et al. 2016 | RCT | Breast cancer, 34 patients | Multidisciplinary team | Mind-body practice (yoga, tai chi, qigong...) | None | ≥ 3 times/week | 3-5 months | Not delivered | NA | Active control group | MFSI-SF | No further outcomes | 3 months | Effective treatment for CRF^†^ |
| van de Wiel et al. 2021 | RCT | Breast and prostate cancer, 137 patients | PT | Combined exercise program (aerobic, resistance, flexibility, stretching, balance…) | Periodic calls | ≥ 3 times/week | 6-11 months | Single initial consultation | NA | Active control group / usual care | MFI | No further outcomes | Not included | Ineffective treatment for CRF |
| Van Waart et al. 2015 | RCT | Breast cancer, 230 patients | Nurse | Aerobic exercise program | None | ≥ 3 times/week | 3-5 months | Single initial consultation | NA | Similar activity, supervised / Usual care | MFI, Fatigue Quality List | Steep Ramp Test | 3 months | Ineffective treatment for CRF |
| VanderWalde et al. 2021 | RCT | Breast cancer, 54 participants | Not specified | Aerobic exercise program | None | ≥ 3 times/week | 1-2 months | Not delivered | NA | Active control group | FSI, PROMIS Fatigue | Short Physical Performance Battery | 1 month | Effective treatment for CRF^†^ |
| Vincent et al. 2013 | Single-arm trial | Breast cancer, 34 patients | PT | Aerobic exercise program | Periodic calls | ≥ 3 times/week | 3-5 months | Not delivered | NA | Not included | PFS-R | 6MWT, VO2 max | Not considered | Ineffective treatment for CRF |
| Vincent et al. 2020 | RCT | Breast cancer, 81 participants | Exercise expert | Combined exercise program (aerobic, resistance, flexibility, stretching, balance…) | Periodic calls | 1-2 times/week | ≥ 1 year | Single initial consultation | NA | Active control group | MFI | 6MWT, VO2 max | Not considered | Ineffective treatment for CRF |
| Wangnum et al. 2013 | RCT | Lung cancer, 60 participants | Multidisciplinary team | Multidimensional program (physical, nutritional, psychological components) | None | Not indicated | 1-2 months | Single initial consultation | NA | Educational group | PFS | VSAQ | Not considered | Effective treatment for CRF^†*^ |
| Wilson et al. 2005 | Single-arm trial | Various with hematopoietic stem cell transplantation, 17 participants | Not specified | Aerobic exercise program | Periodic calls | Not indicated | 3-5 months | Not delivered | NA | Not included | FSI | No further outcomes | Not considered | Effective treatment for CRF^†^ |
| Winters-Stone et al. 2017 | RCT | Breast cancer, 95 participants | Multidisciplinary team | Mind-body practice (yoga, tai chi, qigong...) | None | ≥ 3 times/week | 1-2 months | Not delivered | NA | Educational group | POMS | Self-efficacy for exercise questionnaire, IPAQ-short | Not considered | Effective treatment for CRF^†*^ |
| Yang et al. 2011 | RCT | Breast cancer, 40 patients | Not specified | Aerobic exercise program | Periodic calls | ≥ 3 times/week | 3-5 months | Not delivered | NA | Usual care | MDASI-T | No further outcomes | Not considered | Effective treatment for CRF^†*^ |
| Young Kim et al. 2018 | RCT | Colorectal cancer, 71 participants | Exercise expert | Combined exercise program (aerobic, resistance, flexibility, stretching, balance…) | Periodic calls | Not indicated | 3-5 months | Intensive training during initial period | 3 (25%) | Usual care | FACIT-FS | Godin Leisure-Time Exercise Questionnaire | Not considered | Effective treatment for CRF^†^ |
| Yuen et al. 2007 | Pilot study | Breast cancer, 22 participants | PT | Aerobic exercise program | Periodic calls | ≥ 3 times/week | 3-5 months | Not delivered | NA | Active control group / usual care | PFS | 6MWT | Not considered | Effective treatment for CRF^†^ |
| same as above | Pilot study | Breast cancer, 22 participants | PT | Resistance exercise program | Periodic calls | ≥ 3 times/week | 3-5 months | Not delivered | NA | Active control group / usual care | PFS | 6MWT | Not considered | Ineffective treatment for CRF |
| Yun et al. 2012 | RCT | Various, 273 participants | Not specified | Multidimensional program (physical, nutritional, psychological components) | None | Not indicated | 3-5 months | Not delivered | NA | Usual care | BFI, FSS | ECSI | Not considered | Effective treatment for CRF^†*^ |
| Zhou et al. 2017 | RCT | Ovarian cancer, 74 participants | Exercise expert | Aerobic exercise program | Periodic calls | ≥ 3 times/week | 6-11 months | Not delivered | NA | Usual care | FACT-F | No further outcomes | Not provided | Effective treatment for CRF^†*^ |

Legend: RCT Randomized Controlled Trial, PT physiotherapist; IG Intervention Group; CG Control Group; NA Not Applicable; ^†^ statistical significant difference within-group (compared to baseline values); ^*^ statistical significant difference between-groups (compared to control group).
